# A Note upon Tracheotomy

**Published:** 1907-06-15

**Authors:** 


### A NOTE UPON TRACHEOTOMY.

There is one point in connection with the operation of tracheotomy upon which far too little stress is laid as a rule—and that is the very great *importance of avoiding all injury to the cricoid cartilage.*

Every medical man will have come across one or more of those distressing cases in which, after the performance of tracheotomy in a child for diphtheria or some other condition, it proves very difficult indeed, or even impossible, to leave the tracheotomy tube out. Every attempt to do so, in some cases, results in a suffocating attack of dyspnœa, sometimes at once, sometimes after a few minutes, sometimes even after the breathing has at first been easy and has remained so for some hours. These patients are liable to accidents with their tubes, and now and then, even months after the diphtheria has gone, the child succumbs to such an accident when the nurse is momentarily absent for some cause or other. It is true that many such cases, as they grow older and develop a bigger airway, learn to do without the tube in time but if there is any precaution which can be taken to avoid the difficulty altogether, such precaution should be rigidly observed.

Habituation to breathing through the tracheotomy wound is a potent factor, as is well known, and an attempt to do without the tube is always made before any such habituation is acquired. It is the rule to remove the tube wherever possible within forty-eight hours after tracheotomy for diphtheria. There are cases, however, in which all attempts to follow this good rule fail, and the tube has to be left in week after week. It is often taught that in these cases there is "exuberant granulation tissue" which acts as a constant irritant, and causes laryngeal spasm; and secondary operations are performed for the removal of this granulation tissue. The point we wish to make in this present note, however, is that in nearly every one of these cases of difficulty in leaving out the tube post-mortem examination (should it come to this) shows that the cricoid cartilage has been either notched or, still worse, divided. When one remembers that the two most important pairs of muscles of the larynx are the lateral and the posterior crico-arytænoids, and that these depend for their action upon a firm support from their attachment to the cricoid cartilage, it is little to be wondered at that, if this cartilage is rendered insecure and wobbly by division of its ring, these muscles, acting erratically, close the glottis when they should not. If the erratic action of the two sets of crico-arytænoid muscles were always in the direction of abduction of the cords, the result would not be dangerous; unfortunately adductor spasm always prevails over the abductors, the cause of irritation is not transient, and the patient is asphyxiated unless the tube is reinserted at once.

It takes a long time for a divided cartilage to reunite; repair is very slow; union of its two ends may be many months in coming about, and all this time it will be impossible to leave out the tube. The point is by no means a new one, but it is one which is too little impressed upon us in our student days.

We would repeat, therefore: In performing tracheotomy injury to the cricoid cartilage is one of the most important things to be avoided.

### ALCOHOLISM IN NORMANDY.

M. Raoul Brunon, Professor at the Rouen School of Medicine, has made to the Académie de Médecine an interesting communication regarding "l'alcoolisme de l'enfance." The most striking examples come from Normandy. "It is by no means rare," he says, as reported by *Le Journal*, "to see country women put coffee and brandy in their babies' food. . . . In half the working-class families of Rouen the same stimulants are given to children of from six to eight months old." The effect of the drinking habits of mothers and nurses naturally reacts upon the health of the children. M. Brunon, after condemning the general practice throughout Normandy of adding spirits to coffee, went on to examine the ravages caused among the grown lads throughout the whole of North and Eastern France by absinthe drinking, the weakening of the race being marked among the Norman conscripts by a decrease of height. Within the last twenty years the temperance reform campaign has had an undoubted influence upon the rich and educated classes. "The 'bourgeois' drinks less, while the officer and the student do not drink at all. On the other hand, among the shop assistants and clerks in the working classes and among the peasants alcoholism is spreading, and especially among the women. Hence 'l'intoxication' of the children. The lad and the apprentice are abandoning themelves more and more completely to absinthe. . . . If there is still time, temperance teaching must be organised both in schools and barracks."

